# Mechanism of Disruption of the Amt-GlnK Complex by P_II_-Mediated Sensing of 2-Oxoglutarate

**DOI:** 10.1371/journal.pone.0026327

**Published:** 2011-10-19

**Authors:** Sarah Maier, Paula Schleberger, Wei Lü, Tobias Wacker, Tobias Pflüger, Claudia Litz, Susana L. A. Andrade

**Affiliations:** Institut für organische Chemie und Biochemie, Albert-Ludwigs-Universität Freiburg, Freiburg, Germany; Consejo Superior de Investigaciones Cientificas, Spain

## Abstract

GlnK proteins regulate the active uptake of ammonium by Amt transport proteins by inserting their regulatory T-loops into the transport channels of the Amt trimer and physically blocking substrate passage. They sense the cellular nitrogen status through 2-oxoglutarate, and the energy level of the cell by binding both ATP and ADP with different affinities. The hyperthermophilic euryarchaeon *Archaeoglobus fulgidus* possesses three Amt proteins, each encoded in an operon with a GlnK ortholog. One of these proteins, GlnK2 was recently found to be incapable of binding 2-OG, and in order to understand the implications of this finding we conducted a detailed structural and functional analysis of a second GlnK protein from *A. fulgidus*, GlnK3. Contrary to *Af*-GlnK2 this protein was able to bind both ATP/2-OG and ADP to yield inactive and functional states, respectively. Due to the thermostable nature of the protein we could observe the exact positioning of the notoriously flexible T-loops and explain the binding behavior of GlnK proteins to their interaction partner, the Amt proteins. A thermodynamic analysis of these binding events using microcalorimetry evaluated by microstate modeling revealed significant differences in binding cooperativity compared to other characterized P_II_ proteins, underlining the diversity and adaptability of this class of regulatory signaling proteins.

## Introduction

The survival and growth of an organism in a competitive environment depends on the precise regulation and availability of its natural resources. The essential element nitrogen is assimilated through conversion of nitrate (NO_3_^−^), dinitrogen (N_2_) or a variety of amino acids to ammonium (NH_4_^+^), the only modification of nitrogen that can be readily incorporated into biomolecules. Consequently, reduced ammonium is also a preferred, direct nitrogen source for prokaryotes and plants. The uptake of NH_4_^+^/NH_3_ into the cell is mediated by a family of ubiquitous Ammonium Transport (Amt) proteins that form highly stable trimers within the membrane [1]. Each monomer is composed of eleven or twelve transmembrane helices and a substrate translocation pore with a recruitment site and a selectivity filter [2], [3], [4], [5], [6], [7]. Once in the cytoplasm, ammonium (pK_a_ = 9.25) is readily incorporated into glutamate by glutamate dehydrogenase (GDH) or the ATP-dependent glutamine synthetase (GS). Glutamate∶oxoglutarate amidotransferase (GOGAT) closes this ammonium assimilation cycle by transferring the amido group of glutamine to 2-oxoglutarate (2-OG) to yield two molecules of glutamate [8]. In this process, the action of GS is tightly regulated at both a post-transcriptional and translational levels [9], [10]. Central regulators of GS and Amt are trimeric cytoplasmic proteins of the P_II_ family, termed GlnB or GlnK, respectively [11]. The distinction between the two is not unambiguous and according to current nomenclature *glnK* is the gene that is located in an operon together with the *amt* gene encoding the membrane-integral ammonium transporter, while GlnB is the main regulator of GS and is encoded elsewhere in the genome [11], [12], [13], [14].

P_II_ proteins are key sensors for the metabolic nitrogen status [9], [15], [16], [17]. By direct binding they integrate and respond to the effector molecules 2-oxoglutarate (2-OG) and ATP/ADP, and can be regulated by covalent modification in response to the availability of glutamine, thereby acting as sensors for the cellular levels of carbon, energy and nitrogen, respectively [9], [11], [16], [18].

Prokaryotic and plant P_II_ proteins share a high degree of similarity [19], [20], [21], with a strictly conserved tertiary structure consisting of a four-stranded beta sheet connected by two alpha-helices, and three loop regions of functional relevance. The B-loop, connecting strand 4 to helix II, is important for nucleotide binding, while the extended T-loop between strands 2 and 3 undergoes conformational rearrangements upon effector binding that alter its affinity to the physiological interaction partner and are thus the key element of P_II_ signaling [22]. A third functionally relevant loop region, the C-loop, consists of a carboxyterminal β-hairpin motif that contains two positively charged residues that interact with the phosphate groups of the bound nucleotide. In spite of their highly conserved structure, P_II_ proteins vary strongly in their effector binding kinetics and in the resulting response modulation. They interact with a range of downstream partner proteins, and the underlying, molecular processes are still not fully understood. We are investigating the role of the three GlnK proteins of the hyperthermophilic euryarchaeon *Archaeoglobus fulgidus*, each of which is encoded in a transcriptional unit with a distinct *amt* gene for an ammonium transporter. We have structurally characterized the Ammonium transporter *Af*-Amt1 [2] as well as the P_II_ proteins *Af*-GlnK1 [23] and *Af*-GlnK2 [24]. GlnK proteins, when activated, are sequestered to the cytoplasmic membrane where they bind directly to their corresponding Amt proteins [25], physically blocking ammonium uptake by inserting the T-loops deeply into the transporters' substrate channels [26], [27]. This type of complex formation occurs when the cellular ATP/ADP ratio is low, or when the cellular nitrogen level is sufficiently high, indicated by a low concentration of free 2-OG [28].

The characterization of the binding properties of known effector molecules to these proteins is an essential prerequisite for understanding what factors promote the GlnK-Amt interaction and what are the consequences for metabolic NH_4_^+^/NH_3_ uptake. Our previous work on *Af*-GlnK2 showed a strong and distinct cooperative binding for ATP and ADP, but unexpectedly, no binding of 2-OG [24]. To our knowledge, *Af*-GlnK2 is the first (and so far only) P_II_ protein that is fully insensitive to 2-OG. In the present work we have carried out a comparative analysis of the binding properties of a second GlnK protein from *A. fulgidus*, *Af*-GlnK3, by X-ray crystallography and isothermal titration calorimetry. We find its properties to be more in line with existing data on P_II_ functionality, but with distinct differences in the resulting T-loop conformations that constitute an optimization for their interaction.

## Results and Discussion

### Structural Properties of *Af*-GlnK3

**Figure 1:**
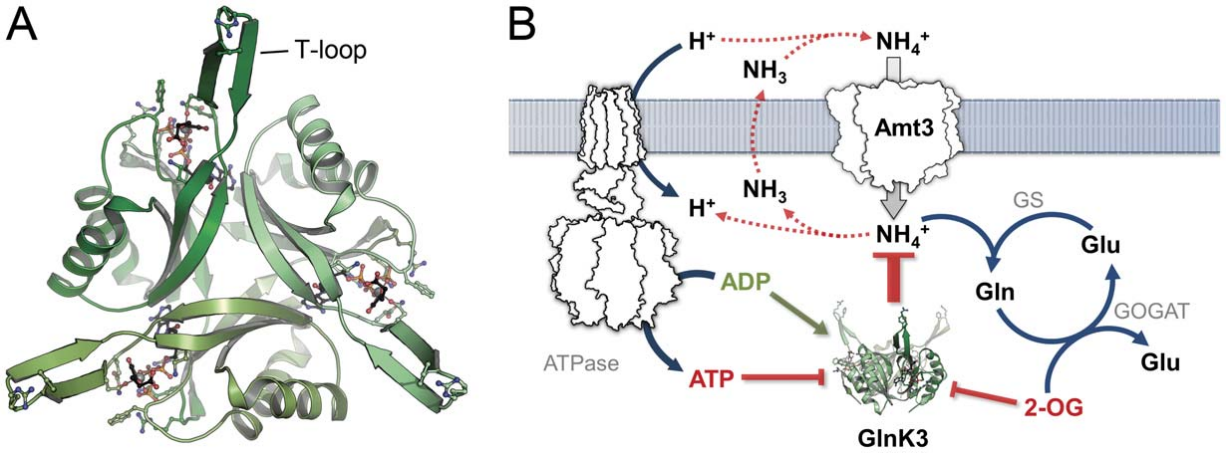
*Af*-GlnK3 and its physiological role in ammonium uptake. **A**) Top view of the trimer *Af*-GlnK3, highlighting the ligand binding sites between the monomers and the protruding T-loops that are required for blocking ammonium transport. **B**) As discussed previously [1], [33], ammonium is actively taken up by Amt proteins and used to aminate glutamate in the ATP-dependent reaction of glutamine synthetase (GS). This reaction is coupled to glutamate∶oxoglutarate amidotransferase (GOGAT) that forms two molecules of glutamate from glutamine and 2-oxoglutarate.

*Af*-GlnK3 shares the canonical fold of P_II_ proteins, consisting of a four-stranded, antiparallel beta sheet and two connecting alpha helices. It forms a tightly packed trimer with approximate dimensions of 30×54 Å (Fig. 1A). The crucial structural feature is the loop connecting beta strands 2 and 3, the T-loop. It spans 21 amino acid residues from G35 to L56 and undergoes conformational changes upon binding of effector molecules that directly affect the affinity of the protein for its interaction partner, *Af*-Amt3. The T-loop shows intrinsic flexibility that is key to its functionality, and in consequence this loop was disordered in most of the P_II_ protein structures available to date [24]. In the present work, the thermostable ortholog *Af*-GlnK3 does allow for the observation of a defined conformation for the T-loop with the bound effectors MgATP and 2-OG (PDB code 3TA2) that prohibits binding to *Af*-Amt3, as well as with bound ADP (PDB code 3TA1), in a conformation that promotes this complex formation.

*Af*-GlnK3 crystallized in the same space group, monoclinic *C*2, without bound ligand (PDB code 3T9Z) and in the presence of either MgATP (PDB code 3TA0) or MgADP (Table 1). The unit cell of these crystals contained two complete trimers of the protein, and – as commonly observed in crystal structures of P_II_ proteins – the T-loop regions were not involved in the formation of crystal contacts. They were partially defined in electron density maps, but showed elevated *B*-factors after refinement as an indication of their flexibility. Only in the ADP-bound state it was possible to model the entire T-loop regions. MgATP and MgADP bound to the protein in a highly conserved nucleotide-binding pocket, a cleft located at the interface of two monomers where the ligands are in direct contact with the B-, C- and the T-loop. The precise modes of ligand binding are highly conserved in all known structures of P_II_ proteins, as is the conformation of the structural core of the trimeric protein itself. Functional differences are largely realized by the actual T-loop conformation that is obtained as a result of ligand binding, and here an immense structural diversity is observed.

**Table 1:** Data collection and refinement statistics.

	value for the indicated crystal type			
Data set	as isolated	MgATP	MgADP	MgATP:OG
**Space group**	*C*2	*C*2	*C*2	*P*6_3_22
**Unit cell constants (Å)**				
***a***	123.4	123.8	123.2	79.2
***b*** ** (** ***β*** **)**	92.8 (133.6°)	93.6 (133.6°)	91.9 (134.3°)	79.2
***c***	88.4	88.6	89.3	223.1
**No. monomers per a.u.**	6	6	6	3
**Resolution range (Å)** *	64.4–1.82(1.92–1.82)	29.44–2.30(2.40–2.30)	64.02–1.90(2.0–1.9)	19.71–1.90(2.0–1.9)
**No. unique reflections**	63,525 (9,300)	32,450 (3,674)	55,158 (8,111)	33,612 (4,666)
**Completeness (%)**	98.2 (98.7)	98.3 (93.4)	98.7 (99.6)	99.8 (100)
**Multiplicity**	3.0 (3.1)	3.5 (3.3)	3.2 (3.3)	16.0 (12.9)
**Mean ** ***I/σ*** ** (** ***I*** **)**	11.0 (3.1)	10.2 (2.3)	10.9 (2.4)	18.0 (4.7)
***R*** **_sym_**	0.048 (0.288)	0.092 (0.363)	0.048 (0.415)	0.135 (0.517)
***R*** **_pim_**	0.034 (0.191)	0.057 (0.229)	0.032 (0.262)	0.034 (0.144)
**No. atoms in model**	4,680	4,735	5,329	3,066
**No. solvent molecules**	353	120	130	389
**Final ** ***R*** **_cryst_**	0.207 (0.236)	0.235 (0.271)	0.212 (0.343)	0.165 (0.192)
**Final ** ***R*** **_free_**	0.236 (0.255)	0.267 (0.328)	0.241 (0.348)	0.197 (0.230)
**r.m.s.d. bonds (Å)**	0.010	0.009	0.009	0.010
**r.m.s.d. bond angles (°)**	1.13	1.19	1.26	1.14
**Mean ** ***B*** ** factor (Å^2^)**				
**protein**	39.8	66.5	62.4	19.5
**water**	50.2	65.5	67.9	32.2
**ligand**	–	54.9	63.7	11.8
**PDB accession #**	3T9Z	3TA0	3TA1	3TA2

*Values in brackets represent the highest resolution shells.

### Control of T-loop conformation

*Af*-GlnK3 was isolated without the addition of nucleotides during protein purification, resulting in a structure without any bound ligand and T-loops that were disordered in the crystal packing. Given that the intracellular concentrations of the nucleotide ligands [29] as well as of free Mg^2+^ are estimated to be rather high [30], it remains doubtful whether this ligand-free structure is of physiological relevance. Without bound ligands, the T-loops were not found to be fixed in a distinct conformation and were consequently disordered in the crystal structure.

As under good nutritional conditions the ATP/ADP ratio in the cell is presumed to be high, the ATP-bound state of *Af*-GlnK3 should represent the common physiological situation, signaling a state of sufficient metabolic energy. Even in crystallization trials with high concentrations of magnesium salts, no Mg^2+^ ions could be unambiguously identified in the binding pocket. Structural variations in the conformation of the three phosphate moieties of the ATP ligand were observed within the asymmetric unit, and the T-loops were still disordered. This situation changed to a uniform conformation with ordered T-loops upon binding of the third known ligand, 2-oxoglutarate. While our previous work on *Af*-GlnK2 yielded the unexpected result that this particular P_II_ family member does not show any affinity for this ligand, 2-OG did bind strongly and specifically to *Af-*GlnK3 (see below), inducing significant structural changes. 2-OG bound to *Af*-GlnK3 with high affinity, but it did so exclusively in the presence of ATP and Mg^2+^. The observed binding mode corresponded exactly to the one described for the P_II_ protein GlnZ from *Azospirillum brasilense* [31], a regulator of nitrogenase activity rather than of an Amt protein. A second P_II_-ATP:2-OG complex was most recently presented for the protein from the cyanobacterium *Synechococcus elongatus* that functionally regulates the activity of *N*-acetyl-L-glutamate kinase as the committed step in arginine biosynthesis [32]. In all cases the binding of 2-OG precludes the formation of an inhibitory complex of the P_II_ protein with its regulatory target and the observed binding mode of the ligand is virtually identical. In the presence of 2-OG, a Mg^2+^ ion is clearly identified in the binding pocket by its near perfect octahedral coordination. Its ligands are three oxo groups from all three phosphates of ATP (thereby discerning ATP form ADP), and one α-carboxy oxygen atom and the α-keto-oxygen of 2-OG. The γ-amido oxygen atom of residue Gln 39 completes the six-fold coordination. This conserved residue is key to the regulatory switch of the protein. It is located directly at the basis of the T-loop, and its actual conformation in *Af*-GlnK3 is dependent on the bound ligand. Under conditions of sufficient energy and nitrogen, both ATP and 2-oxoglutarate levels will be high and the GlnK protein will reside in this blocked state.

**Figure 2:**
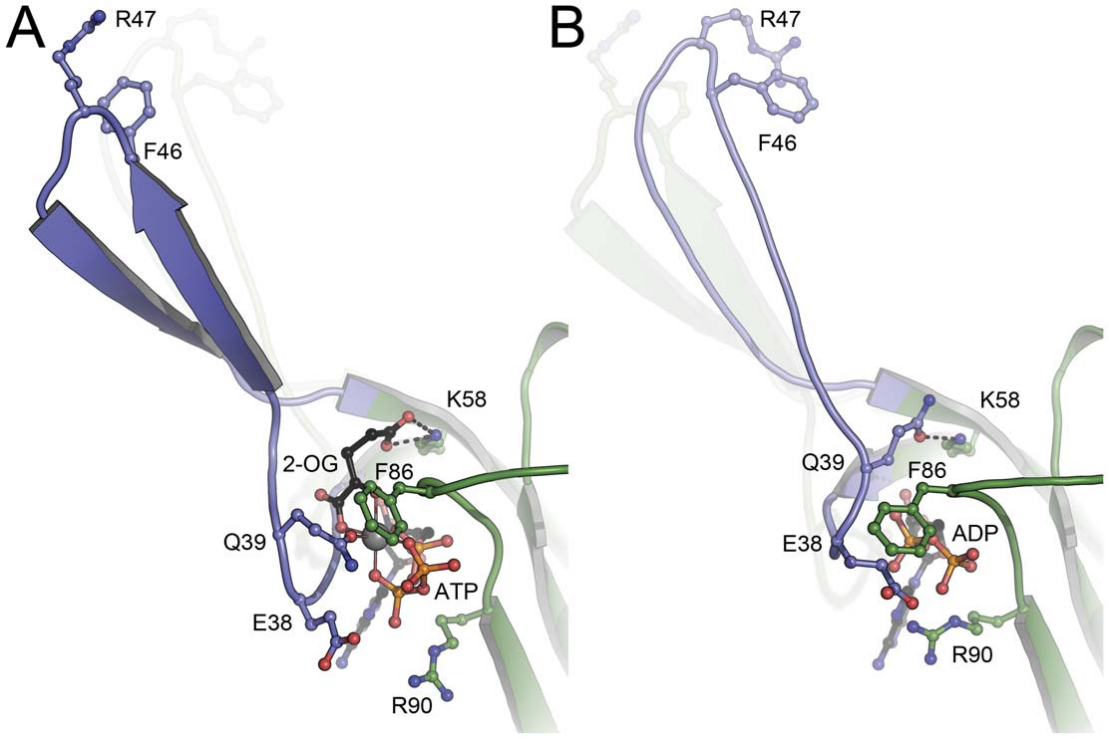
Structural differences between (A) the ATP:Mg^2+^:2-OG complex and (B) the ADP complex of *Af*-GlnK3. In (A) the key ligand 2-oxolutarate requires the presence of ATP for binding and is located at the base of the T-loop (blue), with its ã-carboxy group forming a hydrogen bond to the conserved K58. Residue Q39 is the only protein ligand to the Mg^2+^ ion (grey sphere), and it is this residue that in the ADP complex (B) attains the exact position of 2-OG in (A), forming an analogous hydrogen bond to K58. The resulting tilt and shift of the base of the T-loop leads to a stable â-hairpin structure in (A), compared to a less well-ordered loop in (B) that moves inward by 20°towards the trimer. In both structures, the respective other T-loop conformation is indicated.

A decrease of the cytoplasmic concentration of 2-OG is indicative of either a low carbon status (depletion through kataplerotic reactions) or of a high nitrogen status (conversion to glutamate/glutamine) [28]. In both cases Amt-mediated import of ammonium is no longer desired. *Af*-GlnK3 will return to the ATP-bound state, but will not yet form an inhibitory complex with *Af-*Amt3. Ammonium uptake will continue without negative effects on the cell, unless the energy level of the cell, expressed in the ratio ATP/ADP, starts to drop. At this stage the nucleotide diphosphate will replace ATP as a ligand of the P_II_ protein, and it is this switch that gives the trimeric regulator the competence to bind tightly to *Af-*Amt3 and block transport. Energetic considerations strongly suggest the uptake of ammonium by Amt proteins to be an active mechanism driven by the proton motive force [1], [2]. At the same time, the intracellular accumulation of ammonium is unwanted, as the passive efflux of uncharged ammonia (that is in a protonation equilibrium with ammonium with a pK_a_ of 9.25) would create a futile cycle to degrade the proton gradient [33], [34]. Ammonium is thus swiftly incorporated into glutamate or glutamine, at the expense of one molecule of NADPH or ATP, respectively. In a low energy situation, nitrogen is not required for growth, high-energy metabolites are scarce and the accumulation of intracellular ammonium places further stress on the proton motive force. Consequently, if ATP levels are too low to displace ADP from the GlnK protein, it efficiently shuts off ammonium uptake. In the structure of *Af*-GlnK3 with ADP, key residue Gln 39 was found to point inward to form a short (2.8 Å) hydrogen bond with the side chain of Lys 58 above the nucleotide. At the same time Glu 38 and Lys 101 form a salt bridge at the outward end of the nucleotide binding pocket and Phe 86 in the B-loop closes the remaining gap, effectively sealing up the nucleotide diphosphate within the *Af*-GlnK3 trimer (Fig. 2B). No Mg^2+^ ion was identified in the nucleotide binding pocket in this structure, and the overall conformation was very similar to that of *Escherichia coli* GlnK when bound to the ammonium transporter AmtB [27]. Consequently, this state of *Af*-GlnK3 is the one that is competent to bind to its transporter, *Af*-Amt3.

Upon recovery of the cellular energy status, ADP will once more be displaced by ATP. The bulky γ-phosphate moiety cannot be accommodated without breaking both the salt bridge between Glu 38 and Lys 101 and the hydrogen bond between Glu 39 and Lys 58. In consequence, the base of the T-loop loses its fixation points and the entire region becomes disordered. Whether this state of the protein is competent to associate with the Amt protein remains to be elucidated. While a requirement for the presence of ATP was reported to be a prerequisite to observe complex formation, [35] the available structures of GlnK/AmtB complexes invariably show ADP bound to the P_II_ protein [27]. Structurally, the release of Gln 39 from its hydrogen bond to Lys 58 creates an open binding pocket above the nucleotide that in the ADP complex was occupied by the side chain of Gln 39. Now, however, three oxo groups from the three phosphates of ATP form a pocket for Mg^2+^ and 2-OG, whose binding closes the reaction cycle and leads back to the stable, quaternary complex of *Af*-GlnK3 with ATP, Mg^2+^ and 2-OG. As in *A. brasilense* GlnZ [31], the γ-carboxy group of 2-OG was bound in the same position as the amido group of Gln 39 in the ADP complex.

**Figure 3:**
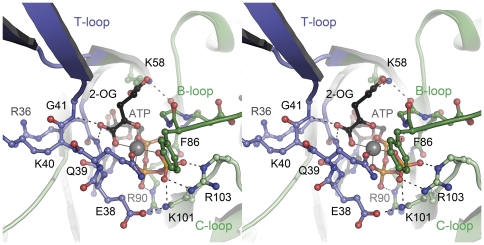
Binding mode of the ligands ATP, Mg^2+^ and 2-oxoglutarate to *Af*-GlnK3. The stereo image shows a view into the ligand-binding cleft located at the interface of two monomers, one of which (dark green) provides the T-loop (blue) and B-loop regions to the binding site, the other monomer (light green) the C-loop. The Mg^2+^ ion (grey sphere) shows octahedral coordination by all three phosphate groups of ATP, by the á-carboxy and á-keto functions of 2-oxoglutarate and by the ã-amido oxygen atom of residue Q39.

**Figure 4:**
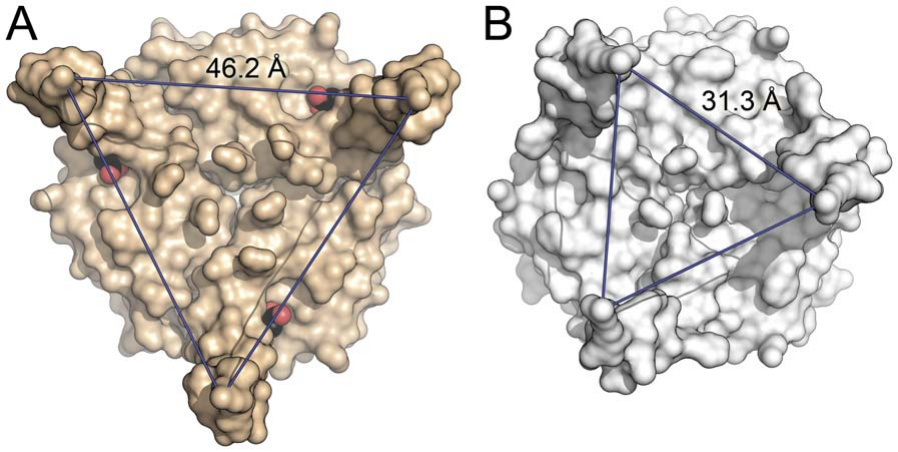
Structural consequences of ligand binding to GlnK proteins. (**A**) With the ligand 2-OG (shown in CPK representation) placed in a wedge-like manner at the base, the T-loops of *Af*-GlnK3 are pried apart in a locked conformation. In the trimer, residues R47 of the monomers are 46.2 Å apart, a distance too large to be able to insert into the substrate channels of the cognate ammonium transporter. (**B**) Structure of the *E. coli* ortholog GlnK as seen in complex with the ammonium transporter AmtB (PDB-ID 2NS1) [27]. The T-loops are ordered and are positioned to fit the substrate channels of the transporter trimer, at a distance of 31.3 Å between residues R47.

Although the binding modes of 2-OG in the three structures available to date are almost identical (and distinct from an earlier observation of a single 2-OG molecule bound to a very different position in *Methanococcus jannaschii* GlnK1) [36], the effect on the conformation of the T-loop is fundamentally different. In the structure of the P_II_ protein from *S. elongatus* the loop is disordered [32], while in *A. brasilense* GlnZ it shows a defined conformation, but points away laterally from the disc-shaped trimer [31]. In *Af*-GlnK3 the T-loops shift to attain a highly ordered β-hairpin conformation stabilized by six hydrogen bonds involving peptide amides, and are fully ordered in the crystal structure (Fig. 2A, 3). Residue Arg 47 that is crucial for insertion into the substrate channels of the Amt protein upon complex formation, remains poised at the apex of the loop. However, 2-OG is fixed at the base of the loop in a wedge-like manner and pushes the T-loops outward with respect to their conformation in the ADP-bound state. In both structures, the C_α_ atoms of residue Arg 47 form an equilateral triangle, but while the sides of this triangle in a complex with the Amt protein have a length of 31 Å, they are extended to 46 Å in the form with bound 2-OG (Fig. 4). They thus lose their structural flexibility and are pried apart too far to be able to insert into the substrate exit channels of *Af*-Amt3. For *A. brasilense* GlnZ, whose direct interaction partner remains to be identified, there is likely no similarly strict requirement and its T-loops, although based on the identical ligand-binding mode, orient differently.

The comparison of the three known complexes of P_II_ proteins with MgATP and 2-OG underlines that the observed binding mode very likely represents the productive and physiological complex of the effectors with all those P_II_ family members that do show affinity to 2-OG. The relevant readout of the P_II_ protein as a signal-processing unit in the prokaryotic and plant cell is the resulting T-loop conformation, and the available structures clearly show that beside the conserved mode of ligand binding, this conformation strongly depends on the respective T-loop itself, i.e. its amino acid sequence. This finding explains why P_II_ proteins, in spite of their highly conserved three-dimensional structures and ligand binding modes, are commonly found to be specific for a single target protein and are not interchangeable.

### Thermodynamics of nucleotide and 2-OG binding

Isothermal titration calorimetry was frequently used in recent studies to investigate the properties of ligand binding to P_II_ proteins [24], [32], [37], [38], [39], and once more the members of this highly conserved protein family revealed striking differences in their ligand binding behavior. The GlnB protein from *S. elongatus* showed binding of ATP, ADP and 2-OG, but did not show any cooperativity [32]. In contrast, *Af*-GlnK2 bound ATP and ADP with distinct negative cooperativity, but was unexpectedly unable to bind 2-OG in the presence or absence of any nucleotide [24]. In the present work we carried out analogous experiments with *Af*-GlnK3, and again the results differ from the previous examples. As are all P_II_ family members studied to date, the protein is insensitive towards glutamine and glutamate, and this is rationalized by the structural data that pointed out that the α-keto group of 2-OG is required for Mg^2+^ coordination, so that its replacement with an α-amino group rules out the observed mode of binding (Fig. 3).

**Figure 5:**
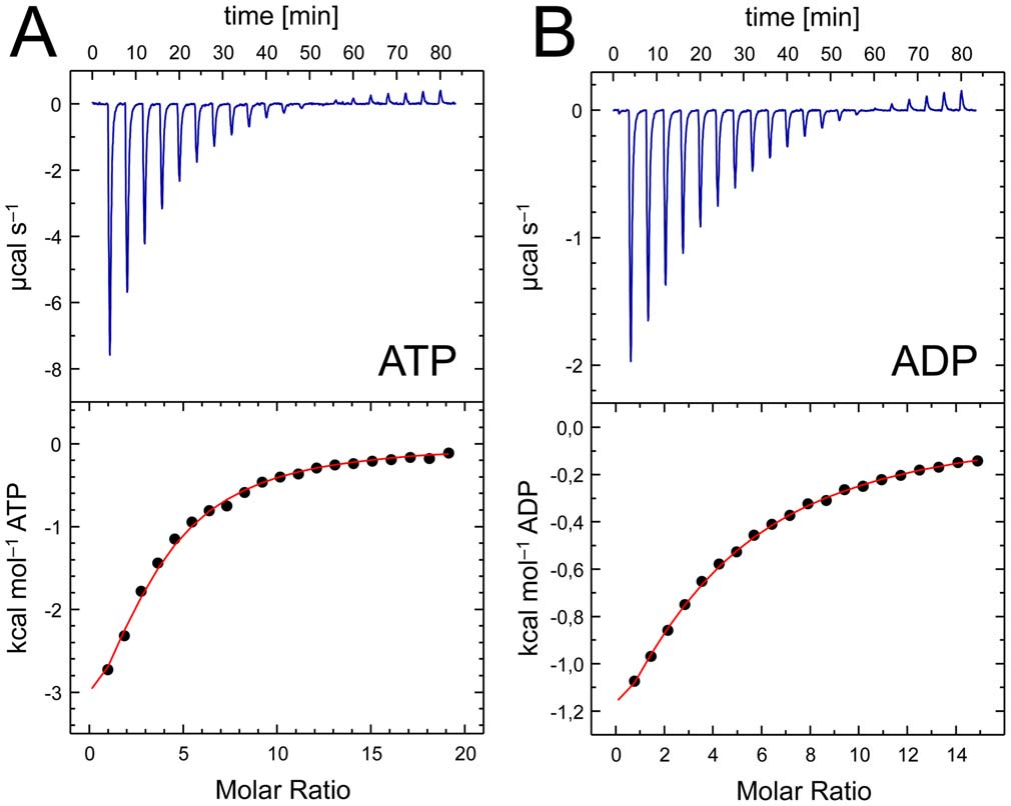
Binding of ATP and ADP to *Af-*GlnK3. Contrary to observations made with the homologous *Af-*GlnK2 the titrations of (**A**) ATP and (**B**) ADP in the presence of 25 mM Mg^2+^ do not show signs of cooperative behavior. The integrated heat data (bottom panel) was fit with a model implying identical sites. The resulting enthalpies and dissociation constants underline that ATP is a much stronger ligand than ADP (Table 2).

**Figure 6:**
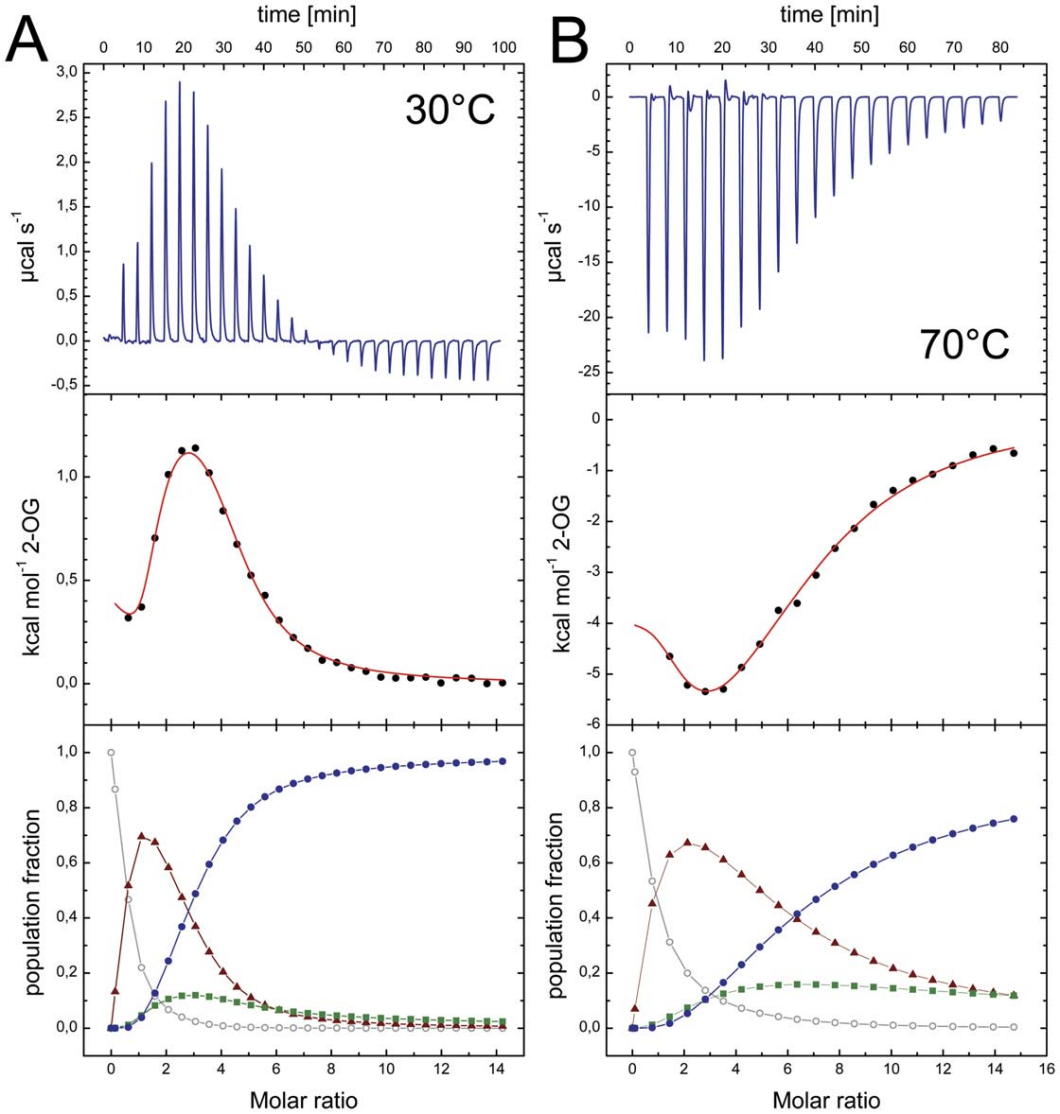
2-OG binding to *Af-*GlnK3 and temperature dependence. **A**) A titration at 30°C shows an initial endothermic event indicating an entropy-driven process that is followed by a strongly exothermic, enthalpy-driven event. In the analysis of population microstates (bottom panel) this translates to an initial accumulation of the singly occupied species (▴) due to negative cooperativity for the second site (▪), but strong positive cooperativity for binding the third ligand (•). Only singly or fully occupied binding sites will be present in relevant amounts. **B**) At 70°C the initial binding event becomes exothermic, leading to a very different overall shape of the experimental curve (top panel). However, analysis of the population microstates shows the same qualitative behavior as in (A).

*Af*-GlnK3 bound MgATP and MgADP, and the binding of 2-OG required pre-incubation of the protein with MgATP, in line with data published on *A. brasilense* GlnZ, *E. coli* GlnK and *S. elongatus* P_II_ [31], [32], [40], [41]. Binding of MgATP to *Af*-GlnK3 was roughly two-fold stronger than binding of MgADP, either at 30 or 70°C although the binding affinity for both nucleotide molecules is clearly higher at 30°C (Table 2). The effect of replacing the bulky F86 for isoleucine, a more common residue among the P_II_ protein family, or proline (as it occurs in *Af*-GlnK2) resulted in a general increase in the nucleotide binding affinities. The F86I variant bound MgATP and MgADP with about 2-fold increased affinity when compared to the wild-type protein, but still displayed a 2-3 fold preference for MgATP binding. Similarly the F86P variant also showed higher binding constants for both nucleotides than the wild-type protein. The affinity constants for MgATP were identical for both variants, but *Af*-GlnK3 F86P showed 5–6-fold higher affinity to MgADP and 1–2 fold stronger binding of MgATP than the wild type (Table 2). The total Gibbs free energy calculations confirm that replacing F86 for an isoleucine resulted in a variant that bound MgATP and MgADP more favorably than the wild type by about 0.5 and 0.3 kcal.mol^−1^, while replacement with a proline resulted in 0.6 and 1.0 kcal.mol^−1^, respectively, changing the nucleotide preference in favor of ADP. When compared to *Af*-GlnK2, the observed affinities for the nucleotides were lower. More importantly, the distinct cooperative binding behavior of the trimeric molecule was absent. The heat developed during the titration experiments of *Af*-GlnK3 with MgATP and MgADP yielded data that could be fit with a single sigmoidal profile derived from a single-site model with three independent sites that did not show cooperativity (Fig. 5, Table 2). However, this was not the case when 2-OG was titrated to *Af*-GlnK3 with bound MgATP. Here the experimental curves showed a complex, cooperative binding scheme that could be fitted using a sequential binding model with three sites. An analysis of the population microstates for 2-OG binding provided the first detailed evidence for the negative cooperativity in this second ligand binding step that is generally assumed for GlnK proteins (Fig. 6) and that allows the proteins to sample a wide range of ligand concentrations.

**Table 2:** Thermodynamic parameters derived from calorimetric titrations of ATP and ADP nucleotides to *Af*-GlnK3.

Ligand	GlnK3	Temp. (°C)	No. binding sites per trimer	Association constantK_A_ (M^−1^)	Dissociation constantK_D_ (µM)	EntalphyΔH(cal·mol^−1^)	EntropyΔS(cal·mol^−1^·K^−1^)	Gibbs Free EnergyΔG (cal·mol^−1^)
**MgATP**	Wild-type	30	2.6±0.2	2960±250	338	−7078±799	−7.5	−4804
		70	3	763±42	1311	−4955±147	−1.2	−4543
	F86I	30	3.0±0.2	6690±824	149	−1115±85	13.8	−5298
	F86P	30	2.87±0.04	7840±307	128	−12000±242	−21.8	−5391
**MgADP**	Wild-type	30	2.8±0.2	1760±89	568	−3538±305	3.2	−4508
		70	3	413±13	2421	−4379±91	−0.8	−4104
	F86I	30	2.1±0.3	2680±230	373	−5730±805	−3.2	−4760
	F86P	30	2.3±0.3	9800±1300	102	−2571±188	9.8	−5542

We further studied the effect of Mg^2+^ on ATP or ADP binding and found that nucleotide binding to *Af*-GlnK3 is far less susceptible to the presence or absence of Mg^2+^ ions than *Af*-GlnK2. Note that in spite of the clear effect of Mg^2+^ on *Af*-GlnK2, the cation was not identified in the complex structures [24]. As these proteins originate from a hyperthermophilic archaeon with an optimal growth temperature of 83°C, calorimetric titrations were also carried out at 70°C, the useful maximum temperature of the calorimeter. These experiments consistently yielded an inferior signal to noise ratio, but did show the same characteristics of binding (Fig. 6B, Table 3). Note that the titration of 2-OG was an endothermic process at 30°C (Fig. 6A) but became exothermic at 70°C (Fig. 6B), in line with thermodynamic expectations. Nevertheless, as evidenced by the population analysis, the mode and type of cooperativity remained unchanged.

**Table 3:** Thermodynamic parameters derived from calorimetric titrations of 2-oxoglutarate to *Af*-GlnK3 pre-incubated with MgATP.

	Wild-type		F86I	F86P
Temp. (°C)	30	70	30	30
**Association constant (M^−1^)**				
**K_A1_**	1.24E5±2.7E4	2.33E4±9.2E3	2.22E5±4.2E4	1.02E4±6.4E2
**K_A2_**	2.62E3±9.1E2	7.67E2±2.9E2	1.40E4±3.5E3	4.09E3±2.6E2
**K_A3_**	3.29E4±9.6E3	4.97E3±1.2E3	2.41E4±4.6E3	2.02E3±5.2E1
**Dissociation constant (µM)**				
**K_D1_**	8.06	429.18	4.50	98.04
**K_D2_**	381.68	1303.78	71.43	244.50
**K_D3_**	30.40	201.21	41.49	495.05
**Free Enthalpy change (cal·mol^−1^)**				
**ΔH_1_**	423±31	−5367±956	−852±60	2005±68
**ΔH_2_**	−2754±1170	−46460±20100	−2838±883	−550±171
**ΔH_3_**	7367±1120	6411±2140	9197±800	13220±147
**Entropy (cal·mol^−1^·K^−1^)**				
**ΔS_1_**	24.7	4.4	21.7	24.9
**ΔS_2_**	6.6	−122.0	9.6	14.7
**ΔS_3_**	45.0	35.6	50.4	58.7
**Gibbs Free Energy (cal·mol^−1^)**				
**ΔG_1_**	−7064.8	−6868.4	−7430.3	−5543.4
**ΔG_2_**	−4739.6	−4595.7	−5748.2	−5006.3
**ΔG_3_**	−6274.75	−5805.1	−6081.8	−4574.9

Values are given for the first, second and third binding event.

### Functional differences within the P_II_ family

The unique inability of *Af*-GlnK2 to bind 2-OG in spite of a high degree of similarity to *Af*-GlnK3 both in amino acid sequence [23] and tertiary structure raises the question for the functional determinants of ligand binding and cooperativity in these proteins. Strikingly, the amino acid residues lining the substrate binding pockets are virtually identical in both cases, and the most obvious discrepancy is found in residue 86, a phenylalanine in *Af*-GlnK1 and *Af*-GlnK3, but a proline in *Af*-GlnK2. In many other orthologs, such as the ones from *E. coli* and *S. elongatus*, this residue is an isoleucine, and in a recent study on revertants for the interaction of *S. elongatus* P_II_ with its effector NAGK, two mutants at this position, I86N and I86T have emerged prominently [32]. Both variants were unable to bind 2-OG, but still showed binding of ATP or ADP. In the aberrant *Af*-GlnK2, residue 86 is a proline, and in order to assess whether this single point mutation was sufficient to abolish binding of the ligand we have created and analyzed the P86F and the F86P variant of *Af*-GlnK2 and *Af*-GlnK3, respectively. In addition we have studied the P86I and F86I variants that mimic the situation in wild type *E. coli* GlnK and *S. elongatus* P_II_ that do not show binding cooperativity for 2-OG.

**Figure 7:**
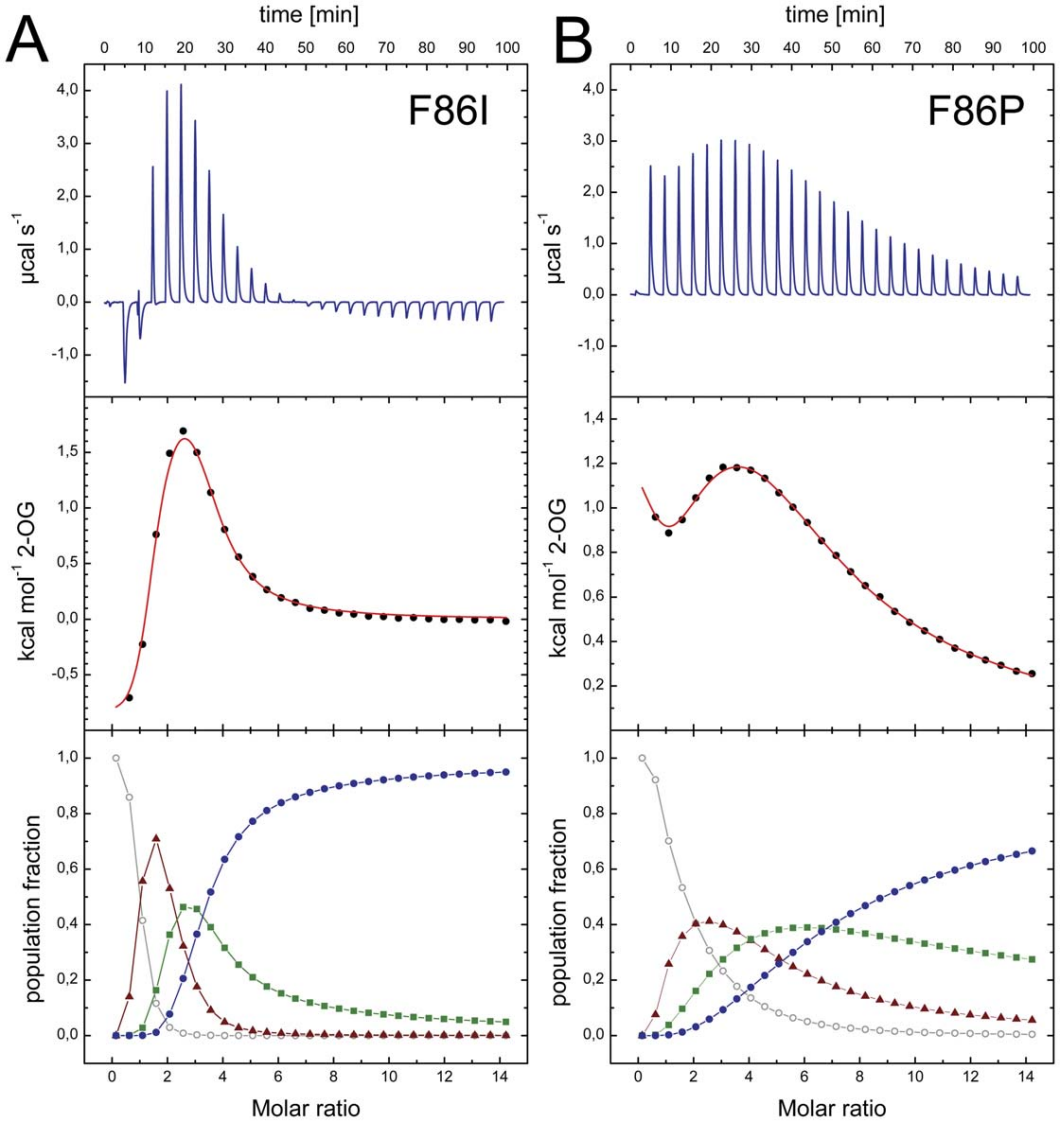
ITC analysis of 2-OG binding to the *Af-*GlnK3 variants F86I and F86P at 30°C. **A**) With respect to the wild type, the F86I variant shows reduced anticooperativity. The population microstate analysis (bottom panel) reveals that initially the singly-occupied species (▴) is populated, but that the negative cooperativity then is weaker so that the state with two bound ligands (▪) does accumulate before it yields to the fully occupied state (•) around a molar ratio of protein *vs.* ligand of 3. **B**) This effect is further enhanced in the F86P variant, where cooperativity is hardly seen in the microstate analysis and the binding sites are occupied sequentially. However, unlike in *Af*-GlnK2 that natively has P86, 2-OG is still bound.

As evidenced by the ITC analysis (Fig. 7) both *Af*-GlnK3 variants retained the ability to bind 2-OG, and in both cases this binding still showed cooperative behavior. The two *Af*-GlnK2 variants however, persisted in their incapacity to bind 2-OG under all tested conditions. Although this disproved our initial hypothesis that residue 86 might be the key to 2-OG binding and cooperativity, the analysis of population microstates did reveal significant differences with respect to wild type *Af*-GlnK3. In the F86I variant, the degree of negative cooperativity in 2-OG binding is reduced (Fig. 7A). The population with two bound ligands – virtually undetectable in the wild type (Fig. 6A) – is significantly populated. In the F86P protein this effect is even more pronounced, to the point that the three sites are sequentially populated and their mutual influence is reduced to a minimum. Residue 86 in the B-loop is thus not essential for 2-OG binding, but it does play a role in tuning the degree of cooperativity that we observe in the different members of the P_II_ family.

### Conclusion

Members of the P_II_ protein family function along conserved lines on the basis of an invariant structural scaffold. They interact with ATP, ADP and 2-OG in a conserved manner, with ligation of ADP acting as an activating signal, while ATP and 2-OG prevent binding to an interaction partner (Fig. 1B). Yet the nature and architecture of this interaction partner are highly variable and diverse, and the available structures point out that this diversity is reached through differences in the conformation of the T-loops in the activated state. This explains why in spite of their structural similarity P_II_ proteins are generally highly specific for their interaction partner. The fine-tuning of binding properties is reached through variations in the sequence of the T-loop itself that, in addition, may or may not contain sites for further regulation through covalent modification. The second important functional property of P_II_ proteins is in the kinetic properties of ligand binding. Different members of the family again show striking variations in cooperativity for the two sequential events of ATP/ADP and 2-OG binding. With the *A. fulgidus Af*-GlnK2 and *Af*-GlnK3 proteins we have now characterized two close orthologs that differ strongly in these properties and shown that these changes likely can not be traced to such simple variations as a F to P mutation in residue 86. Further studies will be required, from which we expect that the *A. fulgidus* GlnK proteins prove to be ideal, stable models for understanding the regulation and optimization of this important class of bacterial signaling molecules.

## Materials and Methods

### Cloning, Overexpression and Purification of *Af*-GlnK3

The *glnk3* gene was obtained from genomic DNA of *Archaeoglobus fulgidus* by PCR using forward (F) and reverse (R) primers carrying the NdeI and XhoI restriction sites, respectively:

(F) 5′- GG**CATATG**AAGATGGTTGTCGCTGTAATAAG - 3′

(R) 5′- GACGGGTGATGAGGAAGTT**CTCGAG**CC - 3′

The PCR product was purified, restriction-digested and ligated into the multiple cloning site of a pre-digested pET21a expression vector (Novagen), yielding a construct with a carboxyterminal pentahistidine affinity tag. The resulting plasmid construct was transformed into *E. coli* BL21(DE3) Rosetta cells (Novagen) for recombinant production. Optimal protein levels were obtained for cells grown at 30°C in Luria-Bertani medium under agitation at 180 rpm. Three hours after induction with 0.1 mM IPTG, cells were harvested by a centrifugation step at 4800 rpm for 15 min at 4°C. Protein was isolated following the protocols established for the orthologs *Af*-GlnK1 and *Af*-GlnK2 [23], [24]. A significant improvement in the final protein yield was obtained after increasing the accessibility of the affinity tag by insertion of three alanine residues (Ala113–115) in the linker between the end of the protein and the pentahistidine tag [23]. This variant was obtained by site-directed mutagenesis using the following forward (and respective reverse) primer:

5′- GGACGGGTGATGAGGAAGTT**GCTGCAGCT**CTCGAGCACCACCACCACCAC-3 ′

SDS-PAGE analysis [42] of pure *Af*-GlnK3 showed a single band corresponding to the monomer of 13 kDa, while gel filtration chromatography allowed for the separation of the trimeric protein peak from a minor fraction of aggregated protein. The buffer used to purify *Af-*GlnK3 was 50 mM Tris-HCl at pH 8.0, supplemented with 300 mM NaCl. Exceptionally, Tris-HCl was replaced by 50 mM HEPES/NaOH at pH 8.0 with the addition of 25 mM MgCl_2_ when purifying protein for ITC measurements at 70°C.

### Crystallization and Diffraction Experiments

The *Af-*GlnK3 trimer fraction from size exclusion chromatography was crystallized by the vapor diffusion method in a sitting drop of 1 µL, containing a 1∶1 mixture of 10 mg/mL *Af-*GlnK3 and a reservoir solution composed of 0.1 M citrate/citric acid buffer at pH 3.5 and 17% (*w/v*) of polyethylene glycol (PEG) 3350. This mixture was equilibrated against 200 µL of reservoir solution and single crystals appeared within one to two days.

Nucleotide-bound (MgATP and MgADP) *Af-*GlnK3 crystals were obtained by soaking with a solution containing 100 mM of the respective nucleotide and 4 mM MgCl_2_, prepared in a buffer solution containing 50 mM Tris-HCl at pH 8.0 and 300 mM NaCl. For cryo-cooling the crystals prior to X-ray exposure, the PEG percentage in the reservoir solution was increased to 27%, together with an extra addition of 5% glycerol.

To obtain crystals of 2-OG-bound to *Af-*GlnK3, a solution of protein was pre-incubated with 2.6 mM ATP in 25 mM MgCl_2_ and 2.6 mM 2-OG were added for co-crystallization. Optimal crystals were formed in a reservoir solution composed of 0.1 M sodium acetate buffer at pH 4.6, 0.2 M NaCl and 30% of 2-methyl-2,4-pentane diol. Crystals were flash-cooled in liquid nitrogen directly from the drop.

Diffraction data sets for the as-isolated and MgADP-bound *Af-*GlnK3 crystals were collected at the Swiss Light Source, SLS, Villingen, Switzerland. GlnK-3 crystals with bound MgATP and MgATP:2-OG were collected on an in-house rotating Cu-anode X-ray generator (Rigaku Micromax 007HF) equipped with a CCD detector (Rigaku Saturn 944+). Data were indexed and integrated using mosflm [43] or XDS [44] in combination with XPREP (Bruker) and scaled with SCALA [43]. Phase information was obtained by molecular replacement with MOLREP [43], using the structure of *Af*-GlnK1 (PDB-ID 3O8W) as a search model. Model building was done in COOT [45] and REFMAC5 [43] was used for refinement. For data collection and refinement statistics see Table 1.

### Isothermal Titration Calorimetry

The calorimetric titration experiments were done according to protocols published previously [24]. Binding of ATP and ADP to the protein in solution was optimized using 0.1–0.13 mM *Af-*GlnK3 (with or without 25 mM MgCl_2_) in the calorimeter cell and 9 mM or 7 mM of the respective nucleotide (with or without 25 mM MgCl_2_) in the titration syringe. All solutions were degassed immediately prior to the experiment, and following a first injection of 2 µL, 21 consecutive injections of 14 µL each were automatically mixed within the stirred assay cell. Temperature variations between the experimental and the reference cells were measured after each injection and recorded as reaction heat *vs*. the molar ratio of ligand to protein, corrected for the successive volume displacement [24]. A 4 min delay between injections was set in the instrument in order to allow re-equilibration of the temperature back to baseline. Different buffers were used for assays at different temperatures: For titration experiments carried out at 30°C, all solutions were prepared in 50 mM Tris-HCl at pH 8.0, 300 mM NaCl with/without 25 mM MgCl_2_ while at 70°C, 50 mM HEPES/NaOH buffer at pH 8.0 with 300 mM NaCl was used.

### Kinetic Simulations

Simulation of the experimental curves recorded for all the ligands that bound to *Af*-GlnK3 was carried out with Origin 7.0 (Microcal) using a simple one-site model for events that did not show cooperative behavior. More complex binding curves were treated as described for the thermodynamic analysis of *Af-*GlnK2, based on an analysis of population microstates [24].

### Protein Data Bank Accession Codes

Structural data were deposited with the Protein Data Bank. Accession codes 3T9Z (as isolated), 3TA0 (MgATP complex), 3TA1 (MgADP complex) and 3TA2 (MgATP:2-OG complex).
